# Transcriptome sequencing and lncRNA-miRNA-mRNA network construction in cardiac fibrosis and heart failure

**DOI:** 10.1080/21655979.2022.2045839

**Published:** 2022-03-02

**Authors:** Shuo Wang, Tianjie Lv, Qincong Chen, Yan Yang, Lei Xu, Xiaolei Zhang, Enmao Wang, Xitian Hu, Yuying Liu

**Affiliations:** Department of Cardiovasology, Shijiazhuang People’s Hospital, Shijiazhaung, HB, China

**Keywords:** Cardiac fibrosis, heart failure, library construction, sequencing, lncRNA-miRNA-mRNA network

## Abstract

Cardiac fibrosis (CF) and heart failure (HF) are common heart diseases, and severe CF can lead to HF. In this study, we tried to find their common potential molecular markers, which may help the diagnosis and treatment of CF and HF. RNA library construction and high-throughput sequencing were performed. The DESeq2 package in R was used to screen differentially expressed mRNAs (DEmRNAs), differentially expressed lncRNA (DElncRNAs) and differentially expressed miRNA (DEmiRNAs) between different samples. The common DEmRNAs, DElncRNAs and DEmiRNAs for the two diseases were obtained. The ConsensusPathDB (CPDB) was used to perform biological function enrichment for common DEmRNAs. Gene interaction network was constructed to screen out key genes. Subsequently, real-time polymerase chain reaction (RT-PCR) verification was performed. Lastly, GSE104150 and GSE21125 data sets were utilized for expression validation and diagnostic analysis. There were 1477 DEmRNAs, 502 DElncRNAs and 36 DEmiRNAs between CF and healthy control group. There were 607 DEmRNAs, 379DElncRNAs,s and 42 DEmiRNAs between HF and healthy control group. CH and FH shared 146 DEmRNAs, 80 DElncRNAs, and 6 DEmiRNAs. Hsa-miR-144-3p, CCNE2, C9orf72, MAP3K20-AS1, LEF1-AS1, AC243772.2, FLJ46284, and AC239798.2 were key molecules in lncRNA-miRNA-mRNA network. In addition, hsa-miR-144-3p and CCNE2 may be considered as potential diagnostic gene biomarkers in HF. In this study, the identification of common biomarkers of CF and HF may help prevent CF to HF transition as early as possible.

## Graphical abstract

**Figure:**
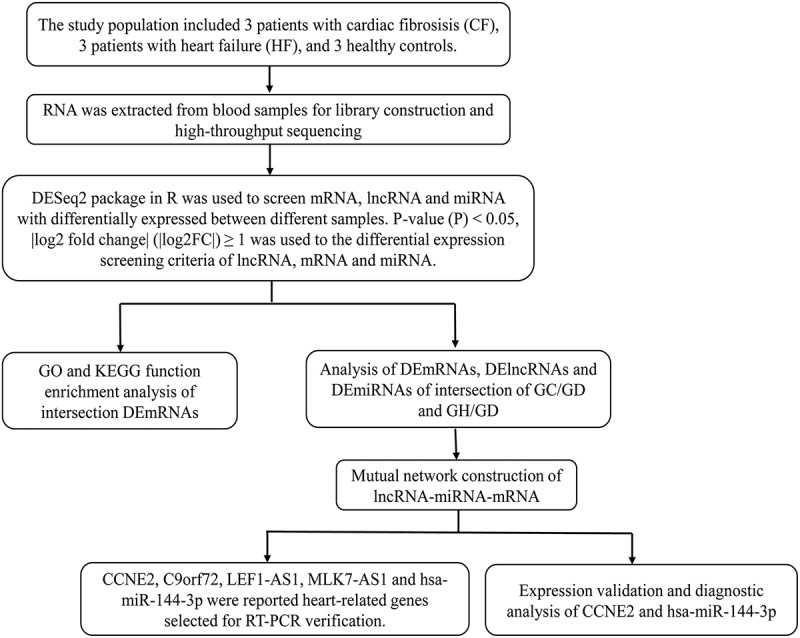

## Introduction

Heart failure (HF), caused by overload and damage to the heart, has a high incidence of morbidity and mortality [1]. Cardiac fibrosis (CF) is often caused by changes in the extracellular matrix (ECM) of the heart, which can change the myocardial structure, promote the development of cardiac dysfunction, induce arrhythmias, and also affect the clinical course and outcome of patients with HF [2,3]. Cardiac fibroblasts are basic cell type that composes the heart and are responsible for ECM homeostasis [4]. CF is defined as a key component of HF and has a strong connection with the progression of HF [5,6].

The interleukin 33 (IL-33) knockout mice showed increased left ventricular hypertrophy, ventricular dilatation, and fibrosis compared with wild-type mice [7]. The miRNAs act as negative regulators of protein translation by affecting mRNA stability [8]. Hsa-miR-223 and hsa-miR-144-3P regulate fibrosis after myocardial infarction by targeting RAS p21 protein activator 1 (RASA1) and phosphatase and tensin homolog (PTEN), respectively [9,10]. Previous studies have shown that low hsa-miR-221 and hsa-miR-222 expression are associated with higher fibrosis and left ventricular hardness in patients with HF [11]. The hsa-miR-197-5p has been shown to be associated with fibrosis in cardiac magnetic resonance imaging of patients with stage C or stage D HF [12]. Interestingly, lncRNA has been found to be an important regulator of heart disease, for example, knockdown of lncRNA-Safe and over-expression of lncRNA-Crnd can alleviate CF [13,14]. These studies highlight that differential expression of mRNAs, miRNAs, and lncRNAs plays an important regulatory role in CF and CH.

Nowadays, gene therapy has become an attractive strategy for the treatment of HF, but there are also many challenges [15]. Histologically, the severity of CF might have been connected with a higher mortality in patients with cardiac diseases, particularly those with HF [16,17]. The detection, prevention, and regression of CF have become important targets for improving HF treatment [2]. Therefore, the analysis of the correlation of gene transcriptome data in CF and HF is of great importance for the later diagnosis and treatment. Previous studies have used transcriptome data to reveal potential biomarkers and developmental mechanisms of CF and HF [18–20]. Transcriptome sequencing is a well-established method for analyzing the entire transcriptome and is commonly used to evaluate the differential expression of genes in case–control studies [21]. However, few studies have used transcriptome sequencing to reveal common potential biomarkers of CF and HF. Thus, in order to determine common potential molecular markers for the early diagnosis and treatment of CF and HF, mRNA, miRNA and lncRNA expression data in CF and HF were analyzed. We selected differentially expressed mRNAs (DEmRNAs), differentially expressed miRNAs (DEmRNAs) and differentially expressed lncRNAs (DElncRNAs) shared by CF and HF to construct the mRNA-miRNA-lncRNA network. Finally, eight candidate molecules (hsa-miR-144-3p, CCNE2, C9orf72, MAP3K20-AS1, LEF1-AS1, AC243772.2, FLJ46284, and AC239798.2) may be used as the diagnosis and treatment targets of CF and HF. Although common biomarkers for CF and HF have been identified, the specific molecular mechanisms between them remain unclear. Identification of key biomarkers provides potential directions for further research. In other words, the identification of common biomarkers of CF and HF may help prevent CF to HF transition as early as possible.

## Material and methods

### Patients

The study population included three patients with CF, three patients with HF, and three healthy controls. All the patients are aged between 51 and 82 years old. Patients who were diagnosed with acute myocardial infarction 1 year later were diagnosed with CF and were included in the CF group. Detailed inclusion criteria for patients with myocardial infarction were as follows: (1) patients have chest pain or distress for >30 min within 24 h, and the myocardial enzymes creatine kinase (CK)-MB (the diagnostic cutoff value used is recommended to be 99th percentile of the upper limit of the reference value for normal people) and cardiac troponin T (cTnT) (within 3–12 h of the onset of acute myocardial infarction, cTnT can rise to 5 to 50 times that of healthy people) were higher than the normal range; (2) patients had a myocardial infarction for the first time; (3) patients did not receive medication or surgery before admission; (4) patients who had blood samples at three time points: before hospitalization, discharged and 6 months after myocardial infarction; (5) patients had complete clinical data, including gender, age, height, weight, etc. The detailed inclusion criteria for patients with HF were as follows: (1) patients must have had HF caused by myocardial infarction; (2) other indicators of patients should meet the diagnostic criteria for HF. Detailed exclusion criteria for patients with CF and HF were as follows: (1)Patients with myocarditis and other diseases caused by chest pain or distress; (2) Patients had a history of renal failure, advanced liver disease, malignancies, and other inflammatory diseases (psoriasis, rheumatoid arthritis, etc.); (3) patients were recurrent; (4) patients who had missing blood samples at three time points: before hospitalization, discharged, and 6 months after myocardial infarction; (5) patient had incomplete clinical data. Healthy controls included people who were considered healthy after a physical examination and did not have any disease.

### RNA isolation

Whole blood samples were taken from each participant. The total RNA was extracted with TRIzol® Reagent [22,23]. The Nanodrop ND-2000 was used for detecting the concentration and purity of the proposed RNA. The integrity of the RNA was confirmed by agarose gel electrophoresis and the RNA integrity number (RIN) value was obtained by Agilent2100 Bioanalyzer [24]. The total amount and concentration of RNA in a single library construction were 5 μg and ≥200 ng/μL, respectively.

## MRNAs and lncRNA library construction, sequencing, and raw data processing

Illumina TruseqTM RNA sample prep kit was used to construct the specific library. Illumina Hiseq X-Ten platform was sequenced using the PE150 strategy [25]. FastQC (http://www.bioinformatics.babraham.ac.uk/projects/fastqc/) [26] was used to check the quality of sequencing and library construction. Fastx-Toolkit (http://hannonlab.cshl.edu/fastx_toolkit/) [27] was used for quality control of raw reads. Specifically, adapter sequence, 5’ segment, 3’ segment, bases with quality <20 and reads with N > 10% were trimmed. The high-quality sequence obtained after quality control was aligned to the human reference genome (GRCh38) in the Ensemble database [28] using the HISAT2 program (https://ccb.jhu.edu/software/hisat2/index.shtml). Expression of mRNAs and lncRNA were normalized and outputted with Stringtie (http://www.ccb.jhu.edu/software/stringtie/) [29]. The abundance of transcripts was measured by fragments per kilobase of exon model per million mapped reads (FPKM).

## MiRNA library construction, sequencing, and raw data processing

The length of 18–30 nt RNA was recovered from the total RNA to construct the small RNAs library. BGISEQ-500 platform was sequenced using the SE50 strategy [30]. FastQC was used to check the quality of sequencing and library construction. Fastx-Toolki was used for quality control of raw reads. Specifically, joint sequence, bases with quality <20 and reads with N > 10% were trimmed. The sequences of length 18–32 nt were extracted. The Rfam (http://Rfam.sanger.ac.uk/) database in blast (http://blast.ncbi.nlm.nih.gov/) was used to annotate the measured small RNA [31]. Clean reads were aligned to the human miRNA precursor and mature miRNA sequences in the miRBase database (http://www.mirbase.org/) [32] using the Bowtie (http://bowtie-bio.sourceforge.net/index.shtml). The secondary structure of sequences (mapped to mature body regions) was predicted through RNAfold (http://rna.tbi.univie.ac.at/cgibin/RNAWebSuite/RNAfold.cgi). The transcription abundance of miRNAs was measured by miRDeep2 (https://github.com/Drmirdeep/drmirdeep.github.io/issues) [33], and the expression level was homogenized by transcripts per million (TPM).

## Differential analysis of mRNAs, lncRNAs, and miRNAs

The DESeq2 package in R was used to screen mRNAs, lncRNAs, and miRNAs with significant differences between different samples [34]. The original read count (mainly to correct the sequencing depth) was standardized. The probability of hypothesis test (P-value) was calculated through the statistical model. Multiple hypothesis testing correction (Benjiamini and Hochberg method) was performed to obtain the corrected p value (false discovery rate, FDR). P-value (P) <0.05, |log2 fold change| (|log2FC|) ≥1 was the screening criteria of DElncRNAs, DEmRNAs, and DEmiRNAs.

## Functional analysis of common DEmRNAs

To research the biological function of common genes, Gene Ontology (GO) [35] and Kyoto Encyclopedia of Genes and Genomes (KEGG) [36] pathway analyses were implemented by using the ConsensusPathDB (CPDB, http://cpdb.molgen.mpg.de/CPDB). P < 0.05 was the screening standard.

## Construction of lncRNA-miRNA-mRNA network

Target mRNAs of common DEmiRNAs were predicted using six miRNA target gene prediction tools (PITA, RNA22, miRmap, microT, miRanda, and PicTar). Only miRNA-target pairs predicted by more than four algorithms can be selected. Then, the predicted target genes were intersected with the common DEmRNAs. The DEmiRNA-DEmRNA targeting and negative correlation pairs were screened out. Diana-lncbase V2.0 was used to predict the targeted relationship between common DEmiRNA and common DElncRNAs [37]. Cytoscape was used to construct the targeted relational network diagram [38]. Subsequently, DEmiRNA in the DEmiRNA-DElncRNA targeted relation pairs and the negative regulatory DEmiRNA-DEmRNA targeted relation pairs were intersects. In the end, lncRNA-miRNA-mRNA network was constructed.

## Real time-polymerase chain reaction (RT-PCR) analysis

The total RNA in the blood samples from three CF patients, five HF patients, and five healthy controls was extracted by TRIzol® Reagent. The RT-PCR analysis sample was inconsistent with the preparation of the library sample. FastQuant cDNA synthesis kit (KR106, TIANGEN) was used for mRNA reverse transcription. MiRNA First Strand cDNA Synthesis kit (Tailing Reaction) (B532451-0020, Sangon Biotech) was used for miRNA reverse transcription. RT-PCR of mRNA and lncRNA was performed using SuperReal PreMix Plus (SYBR Green) SuperReal reagent (FP205, TIANGEN). RT-PCR of miRNA was performed using MicroRNAs qPCR Kit (SYBR Green Method) (B532461-0002, Sangon Biotech). Each experiment was repeated three times. Glyceraldehyde-3-phosphate dehydrogenase (GAPDH), actin beta (ACTB) and hsa-U6 were used as internal control for gene detection. Among them, hsa-U6 is the internal reference of miRNA. Hsa-U6 primers and reverse primer of hsa-miR-144-3P were obtained from the MicroRNAs qPCR Kit. Relative expression levels of genes were calculated using the 2^−ΔΔCt^ method [39].

All experimental procedures were approved by the Clinical Research Ethics Committee of the Shijiazhuang People’s Hospital (2021–019). Written consent was obtained from all the patients.

## Expression validation and diagnostic analysis of key DElncRNAs and DEmRNAs

The GSE104150 (miRNA data from nine cases of HF and seven healthy controls) and GSE21125 (mRNA data from 18 cases of HF and nine healthy controls) data sets were obtained from the Gene Expression Omnibus (GEO) database [40]. We used these two data sets for expression validation and diagnostic analysis of key DElncRNAs and DEmRNAs. The receiver operating characteristic (ROC) analysis was performed by using pROC package in R language. The sensitivity and specificity at the cutoffs were determined according to a previous study [41].

## Statistical analysis

In this study, GraphPad Prism was used for data statistics. For RT-PCR verification experiment, relative expression levels of genes were calculated using the 2^−ΔΔCt^ method. The expression difference of mRNAs, lncRNAs, and miRNAs in CF patient group (GC), HF patient group (GH), and healthy control group (GD) was statistically analyzed by t-test. P < 0.05 was considered as statistical significance.

## Results

HF is caused by overload and damage to the heart. CF is often caused by changes in the ECM of the heart. CF is defined as a key component of HF and has a strong connection with the progression of HF. In order to determine common potential molecular markers for the early diagnosis and treatment of CF and HF, we performed transcriptome sequencing analysis. The study population included three patients with CF, three patients with HF, and three healthy controls. RNA was extracted from blood samples for library construction and high-throughput sequencing. DESeq2 package in R was used to screen DEmRNAs, DElncRNAs, and DEmiRNAs between different samples. Subsequently, functional enrichment analysis, mutual network construction of lncRNA-miRNA-mRNA, in vitro validation, and diagnostic analysis were performed. All results indicate that hsa-miR-144-3p, CCNE2, C9orf72, MAP3K20-AS1, LEF1-AS1, AC243772.2, FLJ46284, and AC239798.2 may be key molecular markers of CF and HF. Hsa-miR-144-3p and CCNE2 may be considered as potential diagnostic gene biomarkers in CF and HF. The identification of common biomarkers of CF and HF can help prevent CF to HF transition as early as possible.

## Differential expression analysis of mRNAs and lncRNAs

**Figure 1.**
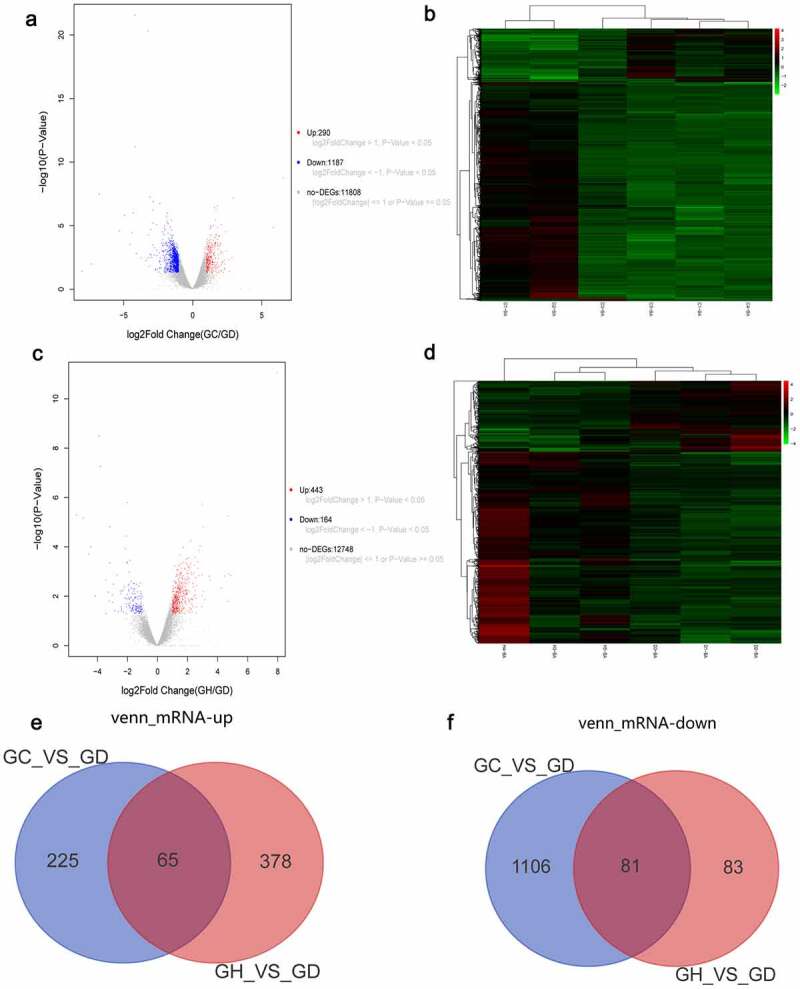
Volcano map, heatmap and venn diagram analysis of DEmRNAs in the GC/GD and GH/GD groups.

There were 1,477 DEmRNAs in the GC/GD group. The volcano map of DEmRNAs is shown in Figure 1a. The heat map of DEmRNAs is shown in Figure 1b. There were 607 DEmRNAs in the GH/GD group. The volcano map of DEmRNAs is shown in Figure 1c. The heat map of DEmRNAs is shown in Figure 1d. In addition, we found 146 common DEmRNAs in GC/GD and GH/GD groups, involved in 65 up-regulated mRNAs (Figure 1e) and 81 down-regulated mRNAs (Figure 1f).

A: Volcano map of DEmRNAs in the GC/GD group; B: Heatmap of DEmRNAs in the GC/GD group; C: Volcano map of DEmRNAs in the GH/GD group; D: Heatmap of DEmRNAs in the GH/GD group; E: Venn diagram of up-regulated DEmRNAs; F: Venn diagram of down-regulated DEmRNAs. In the volcano map, horizontal axis is the value of Log2 logarithmic conversion, and the vertical axis is p-value after logarithmic conversion of -Log10. Heatmap shows result of a two-way hierarchical clustering of DEmRNAs (row) and samples (column). DEmRNAs clustering tree is shown on the left. Red and green represent up-regulated and down-regulated DEmRNAs, respectively. GD, GC, and GH represent the normal control group, the myocardial fibrosis group and the heart failure group, respectively.

**Figure 2.**
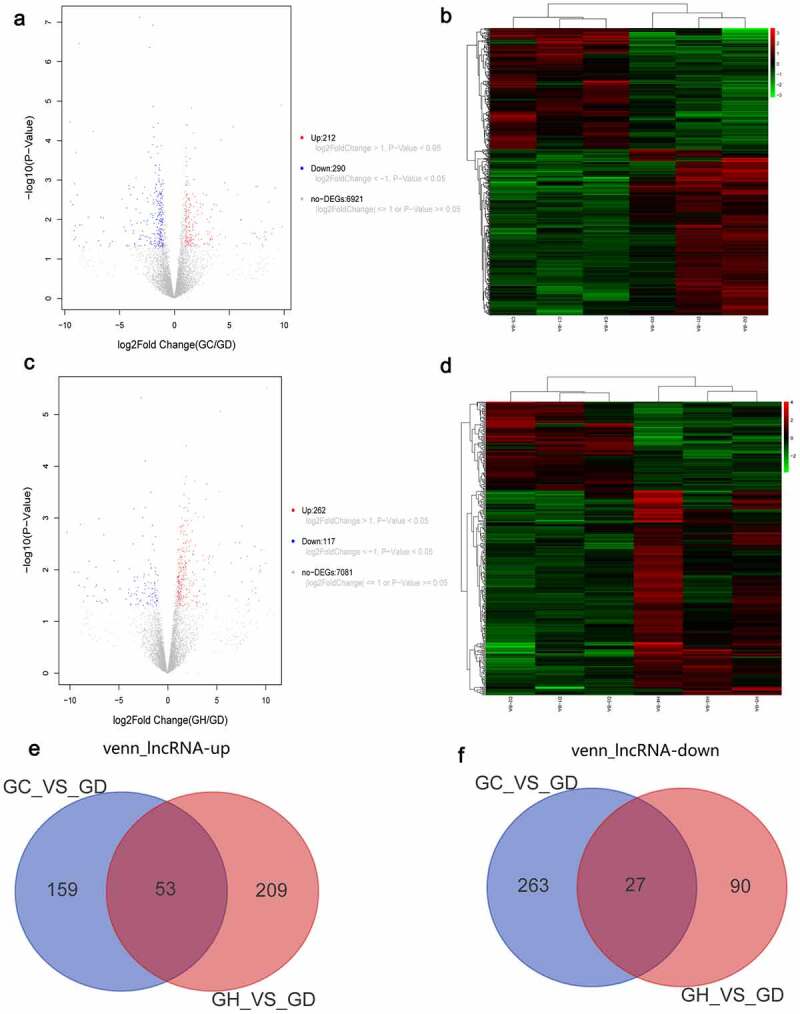
Volcano map, heatmap and Venn diagram analysis of DElncRNAs in the GC/GD and GH/GD groups.

According to the above screening criteria, there were 502 DElncRNAs in the GC/GD group. The volcano map of DElncRNAs is shown in Figure 2a. The heat map of DElncRNAs is shown in Figure 2b. There were 379 DElncRNAs in the GH/GD group. The volcano map of DElncRNAs is shown in Figure 2c. The heat map of DElncRNAs is shown in Figure 2d. In addition, we found 90 common DElncRNAs in GC/GD and GH/GD groups, involved in 53 up-regulated lncRNAs (Figure 2e) and 27 down-regulated lncRNAs (Figure 2f).

A: Volcano map of DElncRNAs in the GC/GD group; B: Heatmap of DElncRNAs in the GC/GD group; C: Volcano map of DElncRNAs in the GH/GD group; D: Heatmap of DElncRNAs in the GH/GD group; E: Venn diagram of up-regulated DElncRNAs; F: Venn diagram of down-regulated DElncRNAs. In the volcano map, horizontal axis was the value of Log2 logarithmic conversion, and the vertical axis is p-value after logarithmic conversion of -Log10. Heatmap shows result of a two-way hierarchical clustering of DElncRNAs (row) and samples (column). DElncRNAs clustering tree is shown on the left. Red and green represent up-regulated and down-regulated DElncRNAs, respectively. GD, GC, and GH represent the normal control group, the myocardial fibrosis group and the heart failure group, respectively.

## Differential expression analysis of miRNAs

**Figure 3.**
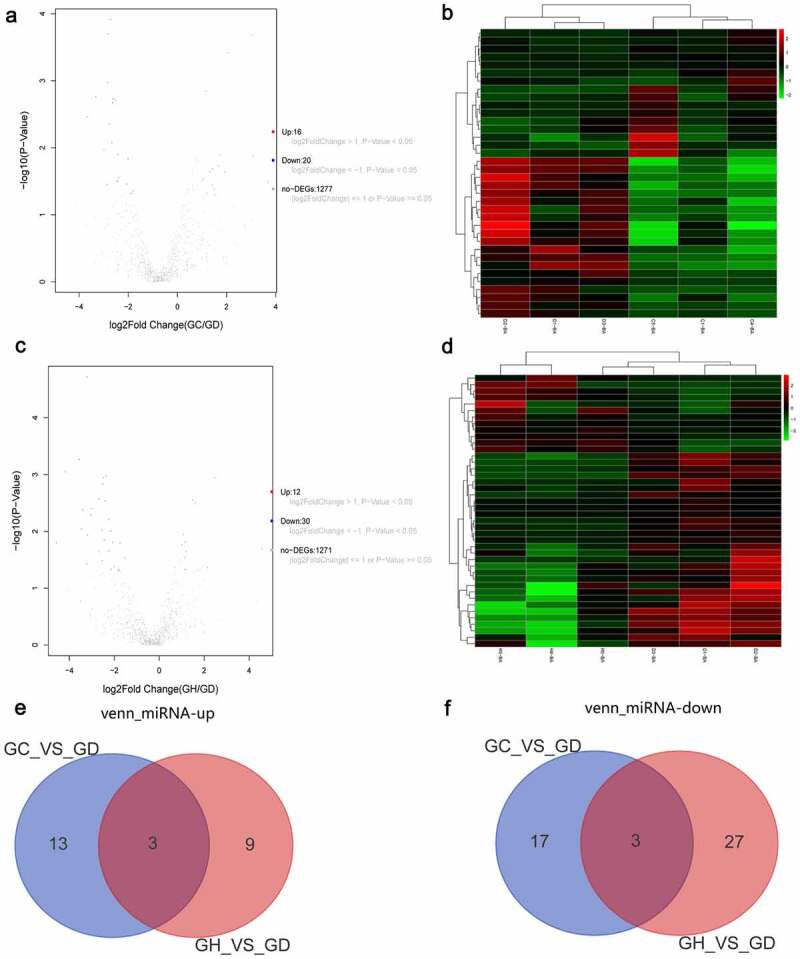
Volcano map, heatmap and Venn diagram analysis of DEmiRNAs in the GC/GD and GH/GD groups.

A total of 36 DEmiRNAs in the GC/GD group were acquired. The volcano map of DEmiRNAs is shown in Figure 3a. The heat map of DEmiRNAs is shown in Figure 3b. A total of 42 DEmiRNAs in the GH/GD group were acquired. The volcano map of DEmiRNAs is shown in Figure 3c. The heat map of DEmiRNAs is shown in Figure 3d. In addition, we found six common DEmiRNAs in GC/GD and GH/GD groups, containing three up-regulated miRNAs (Figure 3e) and three down-regulated miRNAs (Figure 3f).

A: Volcano map of DEmiRNAs in the GC/GD group; B: Heatmap of DEmiRNAs in the GC/GD group; C: Volcano map of DEmiRNAs in the GH/GD group; D: Heatmap of DEmiRNAs in the GH/GD group; E: Venn diagram of up-regulated DEmiRNAs; F: Venn diagram of down-regulated DEmiRNAs. In the volcano map, horizontal axis is the value of Log2 logarithmic conversion, and the vertical axis is p-value after logarithmic conversion of -Log10. Heatmap shows result of a two-way hierarchical clustering of DEmRNAs (row) and samples (column). DEmRNAs clustering tree is shown on the left. Red and green represent up-regulated and down-regulated DEmiRNAs, respectively. GD, GC, and GH represent the normal control group, the myocardial fibrosis group and the heart failure group, respectively.

## Functional analysis of common DEmRNAs

**Figure 4.**
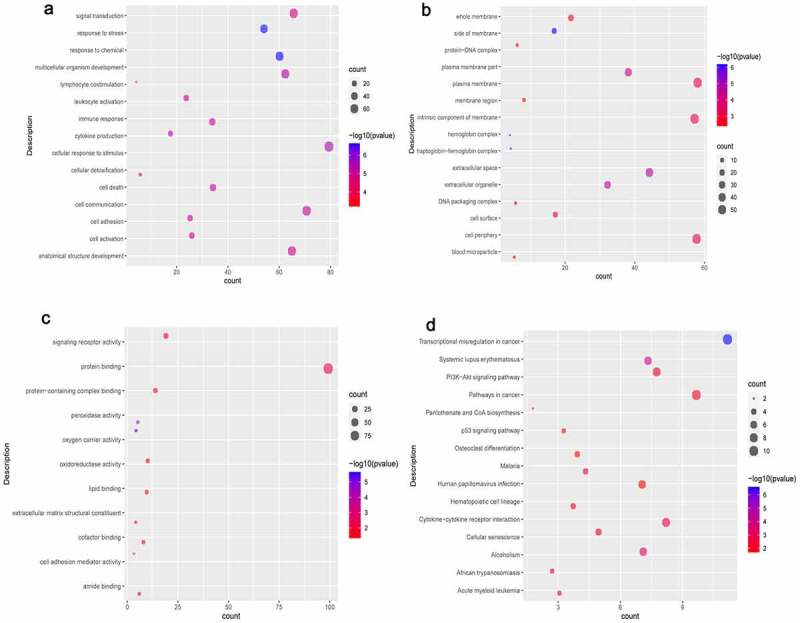
**Significantlly enriched GO terms and KEGG pathways of common DEmRNA**s.

In terms of biological process (BP), DEmRNAs were involved in cellular response to stimulus, cell communication, and signal transduction. In terms of cell composition (CC), DEmRNAs were involved in plasma membrane, cell periphery, and intrinsic component of membrane. BP and CC enrichment results of the top 15 are shown in Figure 4a and 4b. In terms of molecular function (MF), DEmRNAs were involved in protein binding, signaling receptor activity and protein-containing complex binding (Figure 4c). In addition, we found that the DEmRNAs are mainly involved in PI3K-Akt signaling pathway, cellular senescence, and transcriptional misregulation in cancer (Figure 4d).

A: Biological process (BP); B: Cell composition (CC); C: Molecular function (MF); D: Kyoto Encyclopedia of Genes and Genomes (KEGG) pathways. The x-axis shows counts of DEmRNAs enriched in GO terms or KEGG pathways, and the y-axis shows GO terms or KEGG pathways. Red represents up-regulated DEmRNAs and blue represents down-regulated DEmRNAs.

## Mutual network construction

**Figure 5.**
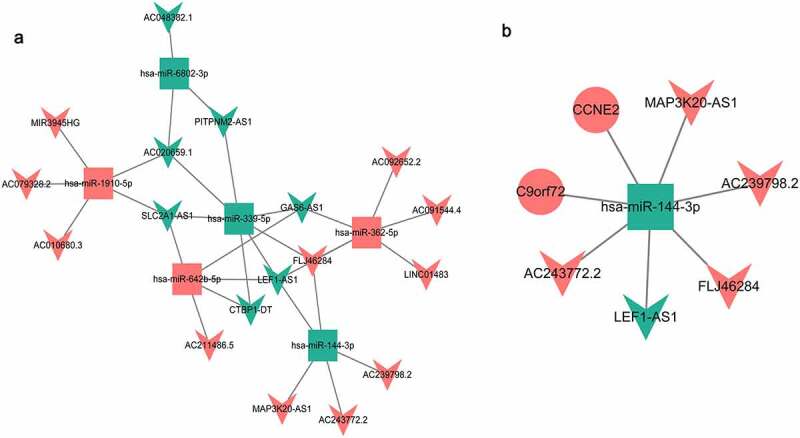
DEmiRNA-DElncRNA targeting network diagram (a) and DElncRNA-DEmiRNA-DEmRNA interaction network (b).

The predicted target genes (Table S1 and Table S2) were intersected with the common DEmRNAs, and the DEmiRNA-DEmRNA targeting and negative correlation pairs were screened out. A total of two DEmiRNA-DEmRNA targeting and negative correlation pairs were obtained (Table 1). In addition, we further verified the targeting relationship of hsa-miR-144-3p-CCNE2/C9orf72 using TargetScan software (http://www.targetscan.org/vert_72/) [42]. The results again confirmed the targeting relationship between hsa-miR-144-3p and CCNE2 (Supplementary Fig. 1). A total of 31 DEmiRNA-DElncRNA targeting correlation pairs were obtained (Table S3). The targeted relationship network between them is shown in Figure 5a. In the end, only one common DEmiRNA was identified in the DEmiRNA-DEmRNA correlation pairs and DEmiRNA-DElncRNA correlation pairs. The lncRNA-miRNA-mRNA network of the DEmiRNA consists of 8 nodes and 7 edges, as shown in Figure 5b.

**Table 1.** DEmiRNA-DEmRNA targeted relationship

DEmiRNA		DEmRNA		
Name	Up/Down	Name	ID	Up/Down
hsa-miR-144-3p	Down	CCNE2	ENSG00000175305	Up
		C9orf72	ENSG00000147894	Up

DEmiRNA: Differentially expressed miRNAs; DEmRNA: Differentially expressed mRNAs; Up: Gene expression levels were up-regulation; Down: Gene expression levels were down-regulation.

Square, circle, V-shape, red and green represents DEmiRNA, DEmRNA, DElncRNA, up-regulation, and down-regulation, respectively.

## RT-PCR validation

**Figure 6.**
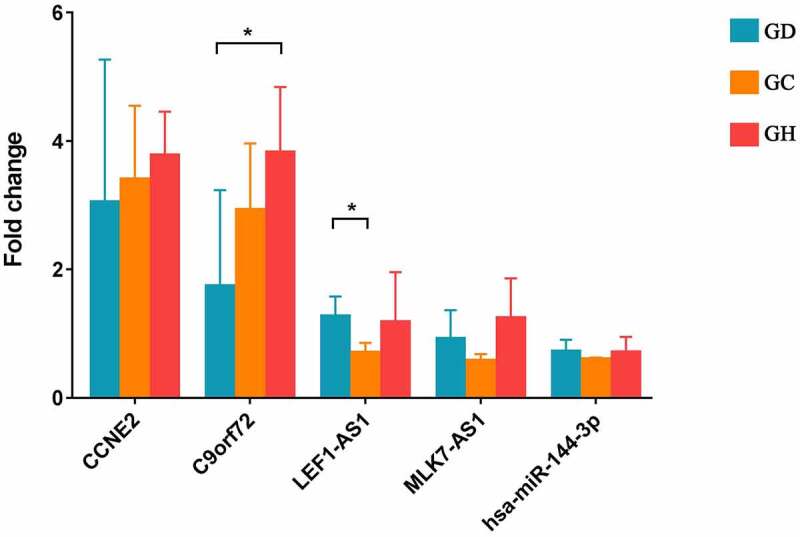
Expression validation of CCNE2, C9orf72, LEF1-AS1, MLK7-AS1, and hsa-miR-144-3p in blood samples by RT-PCR.

In this study, CCNE2, C9orf72, LEF1-AS1, MLK7-AS1, and hsa-miR-144-3p were reported as heart-related genes and selected for RT-PCR verification. The primers are shown in Table 2. Compared with GD, CCNE2, C9orf72 had up-regulation trend and LEF1-AS1, hsa-miR-144-3p were down-regulation trend in GCs. Among which, LEF1-AS1 showed a significant difference. However, MLK7-AS1 had opposite with bioinformatics analysis. Small sample size may cause some errors. Meanwhile, compared with GD, CCNE2, C9orf72, MLK7-AS1 were up-regulation trend and LEF1-AS1, hsa-miR-144-3p had down-regulation trend in GH. Among which, C9orf72 showed a significant difference (Figure 6).

**Table 2.** Primer sequence in the RT-PCR

Primer name	Primer sequence (5’ to 3’)
GAPDH-F (Internal reference)	5-CTGGGCTACACTGAGCACC-3
GAPDH-R (Internal reference)	5-AAGTGGTCGTTGAGGGCAATG-3
ACTB-F (Internal reference)	5-CATGTACGTTGCTATCCAGGC-3
ACTB-R (Internal reference)	5-CTCCTTAATGTCACGCACGAT-3
CCNE2-F	5-AGGAATTGTTGGCCACCTGT-3
CCNE2-R	5-TCCCCAGCTTAAATCAGGCA-3
C9orf72-F	5-TGGGACATGACCTGGTTGC-3
C9orf72-R	5-TCAACGCGGCCAGATAGAC-3
LEF1-AS1-F	5-AGCCGAATTTCCTTAGCCGT-3
LEF1-AS1-R	5-CCACACGTGTTGTGTCAACG-3
MLK7-AS1-F	5-CCTGCAGCACGTTTCCATG-3
MLK7-AS1-R	5-GCCAAATCCAGACCCACCT-3
hsa-miR-144-3p-F	5-TACAGTATAGATGATGTACT-3

GD, GC, and GH represent the normal control group, the myocardial fibrosis group, and the heart failure group, respectively. * represents p < 0.05.

## Expression validation and diagnostic analysis of CCNE2 and hsa-miR-144-3p

**Figure 7.**
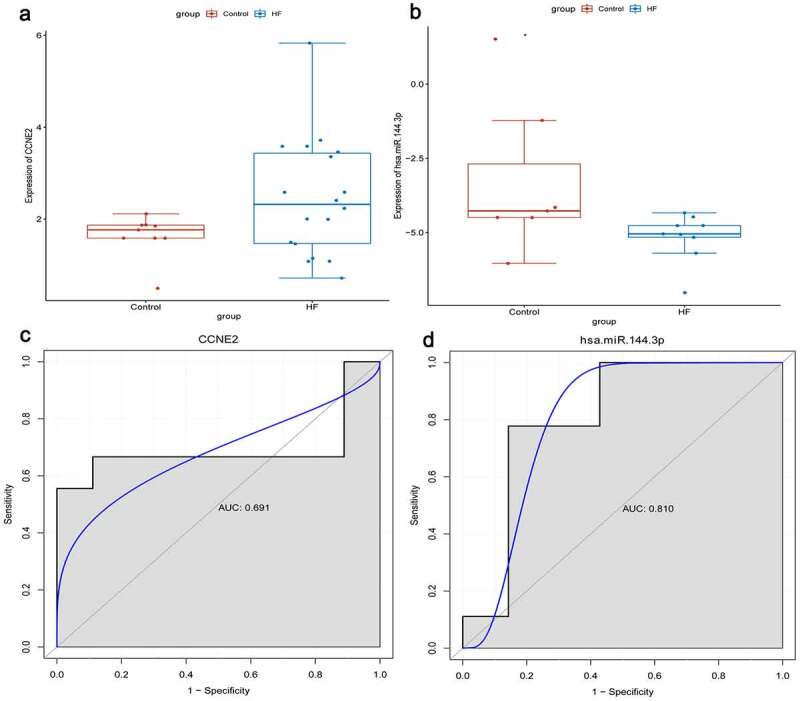
Expression validation and diagnostic analysis of CCNE2 and hsa-miR-144-3p.

RNA sequencing types of GSE104150 and GSE21125 data sets are miRNA and mRNA, respectively. We used these two data sets for expression validation and diagnostic analysis of identified genes. CCNE2, C9orf72, and hsa-miR-144-3p were randomly selected. In our previous analysis, we found that only CCNE2 and hsa-miR-144-3p were expressed in the corresponding data set. We found that the expression levels of CCNE2 and hsa-miR-144-3p were up-regulated and down-regulated in the blood of HF, respectively (Figure 7a, b). This result is consistent with our transcriptome sequencing. Simultaneously, diagnostic analysis of CCNE2 and hsa-miR-144-3p was performed. In the ROC curve analysis, the area under curve (AUC) of CCNE2 and hsa-miR-144-3p were 0.691 and 0.810, respectively (Figure 7c, d). It is indicated that CCNE2 and hsa-miR-144-3p may be considered the potential diagnostic gene biomarkers in HF.

A: Expression validation of CCNE2; B: Expression validation of hsa-miR-144-3p; C: ROC curve of CCNE2; D: ROC curve of hsa-miR-144-3p.

## Discussion

Mechanisms underlying CF and HF progression are mediated by multiple molecules [43–45], and numerous previous studies have been based on molecular studies of a single disease of CF or HF. Conspicuously, HF is invariably accompanied by development of CF [46]. The blood transcriptome can reflect the state of the disease [47,48]. However, few studies have identified common novel biomarkers from the blood transcriptome of CF and HF patients. Therefore, our study aims to identify common molecular markers of CF and HF by analyzing transcriptome sequencing data to provide a potential theoretical basis for studying the transition from CF to HF. In this study, we identified a large number of DEmRNAs, DEmiRNAs, and DEmRNAs, and screened out 146 DEmRNAs, 80 DElncRNAs, and 6 DEmiRNAs shared by CF and HF. Subsequently, eight key molecular markers (CCNE2, C9orf72, hsa-miR-144-3p, MAP3K20-AS1, LEF1-AS1, AC243772.2, FLJ46284, and AC239798.2) were obtained through bioinformatics analysis.

Cyclin E2 (CCNE2) is a cyclin regulating protein that plays a role in G1/S transition in the cell cycle [49]. During heart development, ugly duckling (udu) mutations lead to cardiomyocyte deficiency, which is caused by reduced cell proliferation, and the expression of the cell cycle regulator CCNE2 is reduced during this process [50]. Cardiac fibroblasts are the main cell population of the heart, and an important feature is the ability to produce ECM [51]. Activation and regeneration of fibroblasts are important factors for fibrosis [51–53]. In addition, CCNE2 also promotes cell proliferation and migration [54]. In this study, the expression of CCNE2 was up-regulated. Therefore, we speculated that it might be related to the proliferation and differentiation of cardiac fibroblasts, thus playing an important regulatory role in CF and HF. It is worth mentioning that CCNE2 had a potential diagnostic value for HF in our study.

The protein encoded by the chromosome 9 open reading frame 72 (C9orf72) gene plays an important role in endosomal transport [55]. Interestingly, C9orf72 over-expression can inhibit autophagy in cell starvation [56]. Previous studies have found that the interaction between toll-like receptor 2 (TLR2) and high-mobility group box 1 (HMGB1) participates in the pathogenesis of CF through inhibition of fibroblast autophagy [57]. C9orf72 was an up-regulated gene in this study, so we speculated that it may regulate CF and HF by regulating the autophagy of fibroblasts. Moreover, C9orf72 is also a targeted gene for hsa-miR-144-3p, which further suggests that C9orf72 may play a key regulatory role in the progression of CF and HF.

Hsa-miR-144-3p has been confirmed to play a protective role in ischemia/reperfusion (I/R) by targeting the CUG triplet repeat-binding protein 2- cyclooxygenase-2 (CUGBP-COX2) signaling pathway to reduce I/R-induced cardiomyocyte death [58]. The expression level of hsa-miR-144-3p was markedly down-regulated in myocardial infarction and left anterior descending, compared with normal control [59]. Furthermore, it was confirmed that up-regulation of metastasis-associated lung adenocarcinoma transcript 1 (MALAT1) plays a key role in promoting myocardial apoptosis by targeting hsa-miR-144-3p [59]. In addition, hsa-miR-144 can also improve mitochondrial biogenesis and reduce cell apoptosis by targeting Rac family small GTPase 1 (RAC-1), thereby protecting the heart from hyperglycemic induced damage [60]. In this study, hsa-miR-144-3p was significantly down-regulated, and we speculated that it may participate in the regulation of CF and HF by regulating apoptosis of cardiomyocytes. Moreover, we further verified the targeting relationship of hsa-miR-144-3p-CCNE2/C9orf72 using TargetScan software. The results again confirmed the targeting relationship between hsa-miR-144-3p and CCNE2, which provided a direction for further study of the molecular mechanism of CF and HF. It is worth mentioning that hsa-miR-144-3p had a potential diagnostic value for HF in our study.

Functionally, LEF1 antisense RNA 1 (LEF1-AS1) knockdown can promote cell proliferation and migration [61]. Although LEF1-AS1 has been rarely studied in heart disease, it is up-regulated in many diseases such as cell lung cancer and esophageal squamous cell carcinoma, and promotes cell proliferation [62,63]. MAP3K20 antisense RNA 1 (MAP3K20-AS1) also known as MLK7-AS1, has been mostly studied in cancer, and its reduced expression plays a role in promoting the proliferation of cancer cells [64,65]. In this study, LEF-AS1 and MAP3K20-AS1 were down-regulated and up-regulated, respectively. Therefore, we speculated that LEF-AS1 and MAP3K20-AS1 may be involved in regulating the formation of CF and HF by regulating the proliferation of cardiac myocytes and cardiac fibroblasts. It is noteworthy that the lncRNA-miRNA-mRNA network was formed between hsa-miR-144-3p, CCNE2, C9orf72, LEF1-AS1, MAP3K20-AS1, AC239798.2, AC243772.2, and FLJ46284, which may play an important role in regulating CF and HF through mutual regulation.

GO uses structured vocabularies (or terms) to describe the molecular functions, biological roles, and cellular locations of gene products [66]. In this study, GO enrichment analysis was used to evaluate the potential mechanism of common DEmRNAs. The results showed that these DEmRNAs participated in a variety of biological functions (for example, cell communication and protein binding). Moreover, both cell communication and protein binding are involved in regulating the progression of heart disease [67–70]. In addition, KEGG analysis showed that common DEmRNAs were significantly enriched in PI3K-Akt and cellular senescence signaling pathways. PI3K-Akt and cellular senescence signaling pathways are involved in regulating the occurrence and progression of heart disease via regulating cell survival, apoptosis, growth, cardiac contractility, and even the transcription of related genes [71,72]. The results of GO and KEGG functional analysis suggest that intersecting DEmRNAs may regulate the progression of CF-HF by participating in different biological functions.

Collectively, a number of DElncRNA, DEmiRNAs, and DEmRNAs were identified in this study. However, this experiment has certain limitations. Firstly, the sample size of RT-PCR experiments is small and more blood samples from patients with CF and HF are needed to verify the data of this study. Secondly, the hsa-miR-144-3p network should be functionally validated by experimental evidence in a cell model. Third, the specific molecular mechanism of the identified genes in CF and HF is still unclear, and a large number of experiments are needed to further study their molecular mechanism.

## Conclusion

In this study, 146 DEmRNAs, 80 DElncRNAs, and 6 DEmiRNAs were shared by CF and HF. The identification of hsa-miR-144-3p, CCNE2, C9orf72, MAP3K20-AS1, LEF1-AS1, AC243772.2, FLJ46284, and AC239798.2 in the lcRNA-miRNA-mRNA network implies that they may be involved in the progression from CF to HF. Moreover, according to the ROC analysis results, it is suggested that hsa-miR-144-3p and CCNE2 may be considered as potential diagnostic gene biomarkers in HF. Our study can provide a theoretical basis for the diagnosis and mechanism research of the development process of CF-HF patients and provide potential directions for the latter molecular mechanism research.

## Supplementary Material

Supplemental Material

## Data Availability

The datasets generated during and/or analyzed during the current study are available from the corresponding author on reasonable request. The transcriptome data have been uploaded to Gene Expression Omnibus (accession number GSE196656 and GSE196421).

## Highlights

- Hsa-miR-144-3p and CCNE2 may be potential diagnostic gene biomarkers in HF
- Eight candidate genes may be used as the diagnosis and treatment targets of CF and HF
- Identification of common biomarkers may help prevent CF to HF transition as early as possible
