# Supplementary figures and images for: Transcriptome sequencing and lncRNA-miRNA-mRNA network construction in cardiac fibrosis and heart failure
Source: Bioengineered. 2022 Mar 2;13(3):7118–33. doi: 10.1080/21655979.2022.2045839 (PMC8974171; doi:10.1080/21655979.2022.2045839)

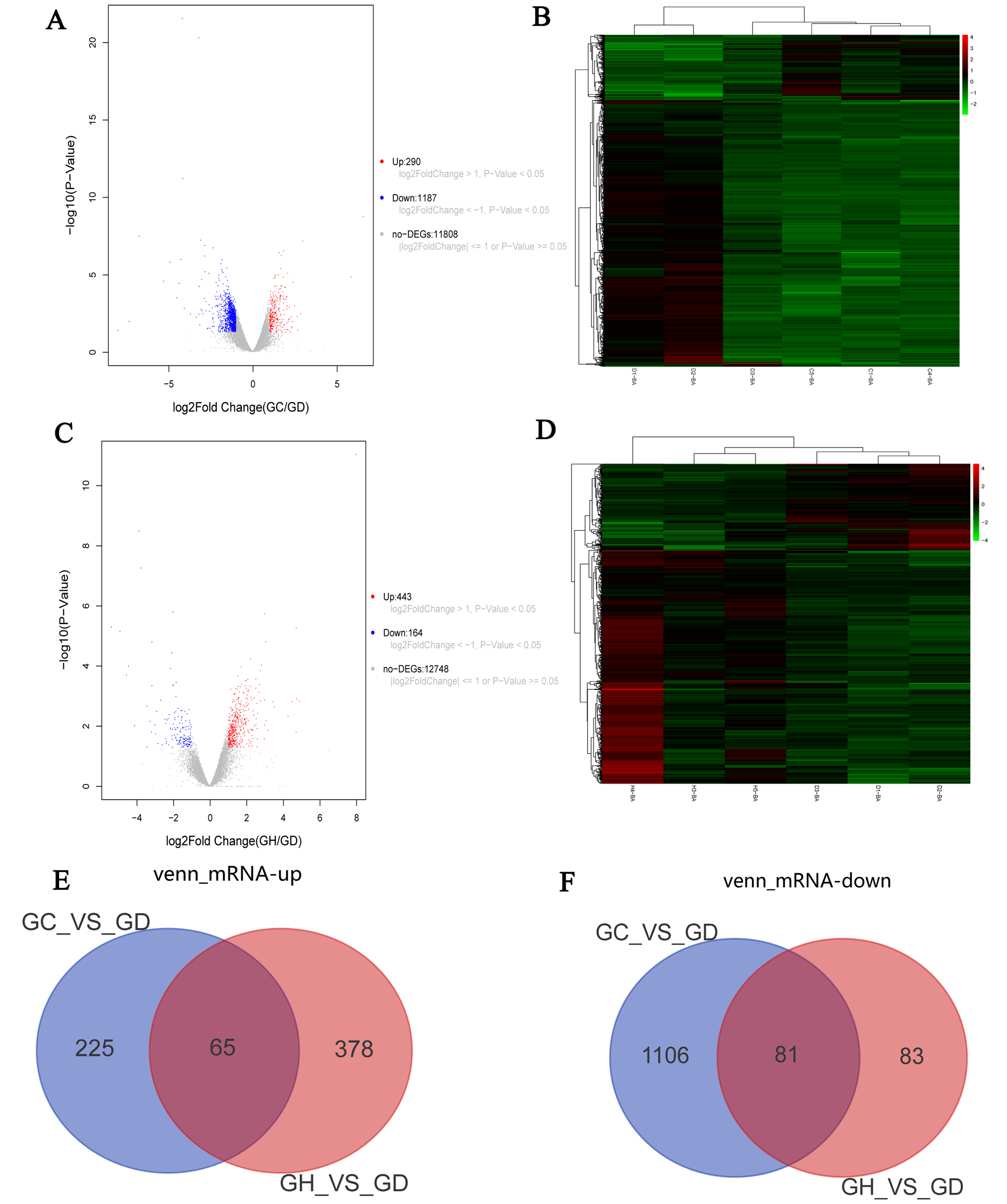

Supplement: Supplemental Material [file KBIE_A_2045839_SM0902.zip › Revision Supplementary Figure 1.tif]
